# Clinical and pathological features of *Nerium oleander* extract toxicosis in wistar rats

**DOI:** 10.1186/1756-0500-7-947

**Published:** 2014-12-23

**Authors:** Tasleem Akhtar, Nadeem Sheikh, Muddasir Hassan Abbasi

**Affiliations:** Cell and Molecular Biology Lab, Department of Zoology, University of the Punjab, Q-A Campus, Lahore, 54590 Pakistan; Department of Zoology, Government College of Science, Wahdat Road, Lahore, 54590 Pakistan

**Keywords:** Histopathology, Inflammation, Toxicity, Polycythemia, Macrocytosis, Neutrophilia

## Abstract

**Background:**

*Nerium oleander* has been widely studied for medicinal purposes for variety of maladies. *N. oleander* has also been reported having noxious effects because of its number of components that may show signs of toxicity by inhibiting plasma lemma Na^+^, K^+^-ATPase. The present study was performed to scrutinize the toxic effect of *N. oleander* leaves extract and its clinical and pathological features in wistar rats.

**Results:**

Hematological analysis showed significant variations in RBCs count (P = 0.01), Hb (P = 0.001), Hct (P = 0.0003), MCV (P = 0.013), lymphocyte count (P = 0.015), neutrophil count (P = 0.003), monocyte count (P = 0.012) and eosinophil count (P = 0.006). Histopathological studies have shown that in T_1_ group noticeable infiltration of inflammatory cells was found with low level of vascular damage. In T_2_ group*,* increased proportion of binucleated and inflammatory cells, hepatic necrosis, widening of sinusoidal spaces and mild level of vascular damage was observed.

**Conclusion:**

Taken together these findings we can conclude that *N. oleander* leaves extract significantly affects on experimental animals due to its toxicity. Efforts must be exerted to purify different chemical components from extract with no inflammation as this plant is utilized in folk medicine with narrow therapeutic indices.

## Background

*Nerium oleander* commonly known as “Kaner”, belongs to the family Apocynaceae. It is native to Indo-Pak subcontinent, widely distributed in Mediterranean region, subtropical Asia, southern United States and many other warms areas [1] where it grows outdoors in parks, gardens and along roadsides by people who may not consider their toxic potential [2].

All parts of the plant are reputed as therapeutic agents and have been used in folklore in a variety of ailments including skin complaints, ringworm infections, opthalmia, cancer, epilepsy, eczema, malaria and gastrointestinal disturbances. Leaves and bark are also used as heart tonic, antibacterial, diuretic and anti-emetics [3–7].

On the contrary *N. oleander* has been regarded as poisonous plant due to a number of its components that may show signs of toxicity. Toxic exposure of humans and different species of domestic animals to *N. oleander* cardenolides occurs commonly throughout the geographic regions where this plant grows [8]. The human mortality associated with ingestion of oleander is generally very low, but animals exposed to the plant are often found suddenly dead owing to cardiac dysfunction. *N. oleander* contains a mixture of very toxic cardiac glycosides of cardenolides, the most prominent of which are oleandrin and neriine [9, 10]. Cardiac glycosides of *N. oleander* cause poisoning by inhibiting plasmalemmal Na^+^, K^+^-ATPase [11]. The plant has also been used for suicidal or murderous intention [12]. Accidental and/or experimental *N. oleander* toxicities have been reported in cattle [13], horses [14], sheep [8], goats [9], donkeys [15], camels [16], cats [17], dogs [18], monkeys [19], budgerigars [20], geese [21], ducks [22], turkeys [23], toed sloths [24] and bears [9].

The intention of this study was to determine the toxic nature of *N. oleander* leaves extract and its clinical and pathological features in Wistar rats.

## Methods

### Animals

Healthy Wistar rats (about 175 ± 25 g) were arranged from the department of Zoology, University of the Punjab Lahore, housed in wire-bottomed cages in an animal room under standard conditions with 12-h light/dark cycles and at an ambient temperature of 22 ± 1°C, with fresh water and food pellets available *ad libitum* in compliance with the institutional guidelines and the study was approved by the ethics committee of the department of Zoology, University of the Punjab Lahore.

### Dose preparation & administration

*N. oleander* leaves extract was prepared by boiling air-dried leaves in 0.9% NaCl solution (1:1, w/v) for 3 h by steam distillation. The extract was then filtered and used to the experimental animals. Rats were alienated into three groups, designated as Con group for control animals and T_1_ & T_2_ groups for experimental animals. T_1_ & T_2_ groups were given with *ad libitum* access to *N. oleander* leaves extract for 3 and 7 days respectively, whereas Con group was given with normal drinking water.

### Blood & liver tissue sampling and processing

All the animals were anesthetized with intra-peritoneal injection of ketamine – distilled water mixture (1:1), (50 mg/ml of ketamine) and scarified. The dissections were done in aseptic maintained conditions to draw the blood through direct cardiac puncture and excise the liver out. The blood samples were collected in sterilized disposable syringes (Becton Dickinson, Private Ltd.), 2 ml of the blood was transferred to K3-EDTA coated vacutainers (Becton Dickinson, Private Ltd.) for complete blood count and liver of each animal, obtained after dissection was placed in Petri dish containing 0.9% saline, cut into 1 × 1 cm pieces and stored with 10% formalin in labeled glass bottles until.

### Evaluation of hematological parameters

Complete blood counts were performed on the samples of control and treated animals, using an automated blood cell analyzer Sysmex XT-1800i (Japan). It utilizes the technology of fluorescent flow cytometry and hydrodynamic focusing. Fluorescent technology consistently differentiates normal white blood cells (WBCs), RBCs and platelets (plts) from abnormal populations, thereby decreasing the number of manual interventions.

### Evaluation of histopathological variations

Small pieces of liver fixed in 10% formalin were dehydrated in ethanol (40%-100%), cleared in xylene and embedded in paraffin wax. Sections (5 μm thick) were cut and stained with haematoxylin and eosin and examined for histopathological changes under the microscope (Olympus VANOX). The microphotographs were taken using digital camera (Nikon SLR-D3000) camera at original magnification of 100X & 1000X.

### Statistical analysis

The data were analyzed using Prism Graph pad 5 software (San Diego, CA). Statistical significance was calculated by means of one-way ANOVA test pursued by Tukey’s post hoc analysis to assess differences against the control group and p-values less than or equal to 0.05 were thought-out significant.

## Results

### Consequence of *N. oleander*leaves extract on Red blood cells count & Red blood cell indices

**Figure 1:**
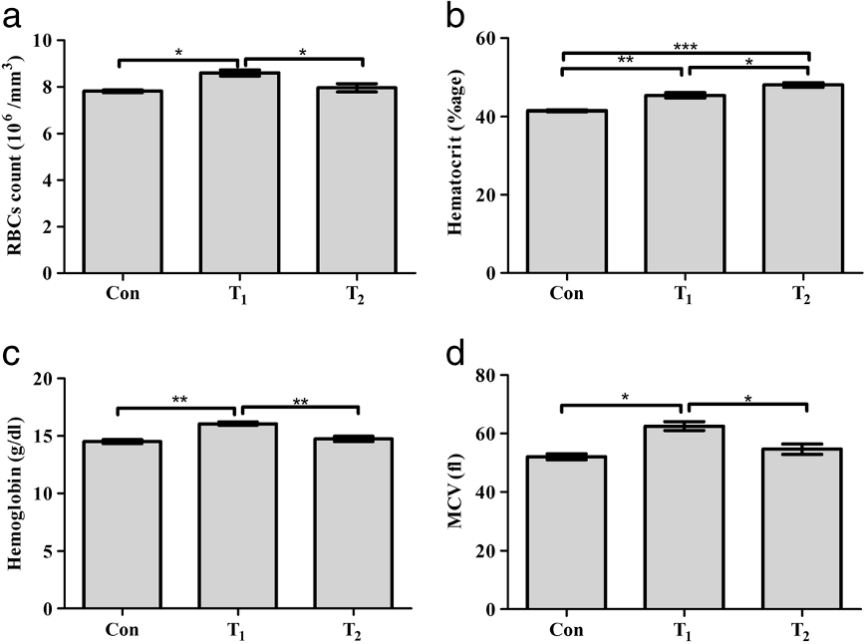
**Hematological changes in the control and experimental groups.** **a**: Effect of *N. oleander* leaves extract on RBCs count; **b**: Effect of *N. oleander* leaves extract on hematocrit level; **c**: Effect of *N. oleander* leaves extract on hemoglobin level; **d**: Effect of *N. oleander* leaves extract on MCV; Con (control group); T_1_ (*N. oleander* oral intake for 3 days) and T_2_ (*N. oleander* oral intake for 7 days). Values are mean ± SEM, error bar indicating the standard deviation. * = P ≤ 0.05, ** = P ≤ 0.01, *** = P ≤ 0.001.

Blood level of Red blood cells (RBCs) reflects a statistical significant increment of 11% in T_1_ group with reference to con group while T_2_ group shows more or less a steady level of RBCs with minor increment (P = 0.011) (Figure 1a). Hematocrit (Hct) contents show a statistical significant increase of 10% & 16% respectively in T_1_ & T_2_ groups as compared to con group (P = 0.0003) (Figure 1b) while hemoglobin (Hb) & mean corpuscular volume (MCV) reflect a significant increasing trend with 11% & 20% increment in T_1_ group in comparison with con group. At the same the T_2_ group shows minor increase of Hb & MCV, 2% & 5% respectively as compared to con group (P = 0.001 & 0.013) (Figure 1c,d and Table 1).

**Table 1:** **Effect of** ***N. oleander*** **leaves extract on Red blood cells count & Red blood cell indices (Mean ± SEM) Con, control; T**_**1**_ **,** ***N. oleander*** **oral intake for 3 days; T**_**2**_ **,** ***N. oleander*** **oral intake for 7 days**

	RBCs	Hct	Hb	MCV
Con	7.810 ± 0.055	41.430 ± 0.185	14.500 ± 0.152	52.000 ± 1.000
T_1_	8.600 ± 0.125*	45.330 ± 0.666**	16.070 ± 0.145**	62.500 ± 1.500*
T_2_	7.960 ± 0.181	48.000 ± 0.577***	14.730 ± 0.218	54.670 ± 1.764

Data were processed by one-way ANOVA followed by Tukey’s *post hoc*: *P < 0.05, **P < 0.01 and ***P < 0.001.

### Consequence of *N. oleander*leaves extract on differential leukocyte count

**Figure 2:**
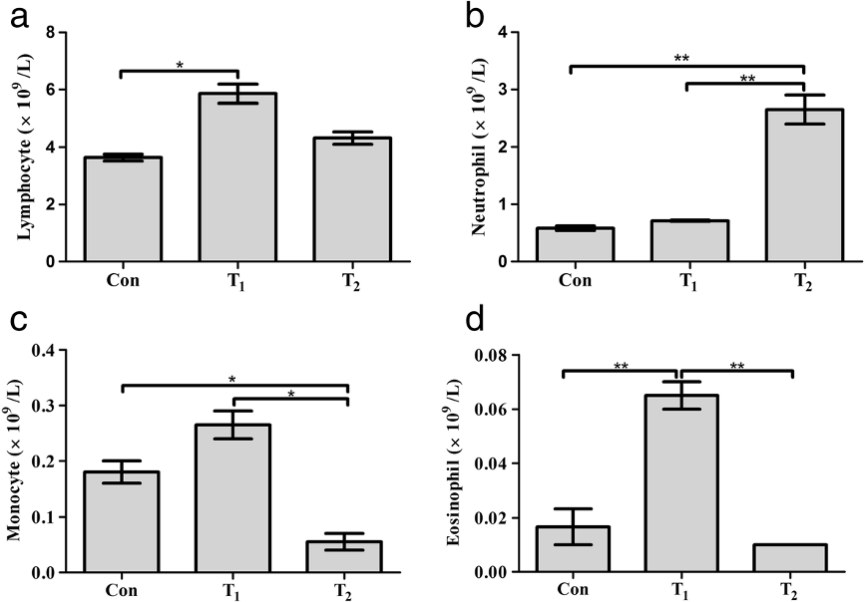
**Differential leukocyte changes in the control and experimental group.** **a**: Effect of *N. oleander* leaves extract on lymphocyte count; **b**: Effect of *N. oleander* leaves extract on neutrophil count; **c**: Effect of *N. oleander* leaves extract on monocyte count; **d**: Effect of *N. oleander* leaves extract on eosinophil count; Con (control group); T_1_ (*N. oleander* oral intake for 3 days) and T_2_ (*N. oleander* oral intake for 7 days). Values are mean ± SEM, error bar indicating the standard deviation. * = P ≤ 0.05, ** = P ≤ 0.01, *** = P ≤ 0.001.

A rise of 61% lymphocyte count in T_1_ group and 19% in T_2_ group with reference to the respective con group was observed (P = 0.015) (Figure 2a). Neutrophils exhibited a marked statistical increase of 357% in T_2_ group and a slight increase of 22% in T_1_ group concerning con group (P = 0.003) (Figure 2b). Monocytes and eosinophils showed an increase of 47% & 290% respectively as compared to con group in T_1_ group, while a decline of 70% & 40% respectively was noted in T_2_ group (P = 0.012 & P = 0.006 respectively) (Figure 2c,d and Table 2).

**Table 2:** **Effect of** ***N. oleander*** **leaves extract on differential leukocyte count (mean ± SEM) Con, control; T**_**1**_ **,** ***N. oleander*** **oral dose for 3 days; T**_**2**_ **,** ***N. oleander*** **oral dose for 7 days**

	Lymphocyte count	Neutrophil count	Monocyte count	Eosiniphil count
Con	3.635 ± 0.115	0.580 ± 0.040	0.180 ± 0.020	0.016 ± 0.006
T_1_	5.860 ± 0.330*	0.710 ± 0.010	0.265 ± 0.025	0.065 ± 0.005**
T_2_	4.315 ± 0.215	2.650 ± 0.250**	0.055 ± 0.015*	0.010 ± 0.000

Data were processed by one-way ANOVA followed by Tukey’s *post hoc*: *P < 0.05 and **P < 0.01.

### Histopathological analysis

**Figure 3:**
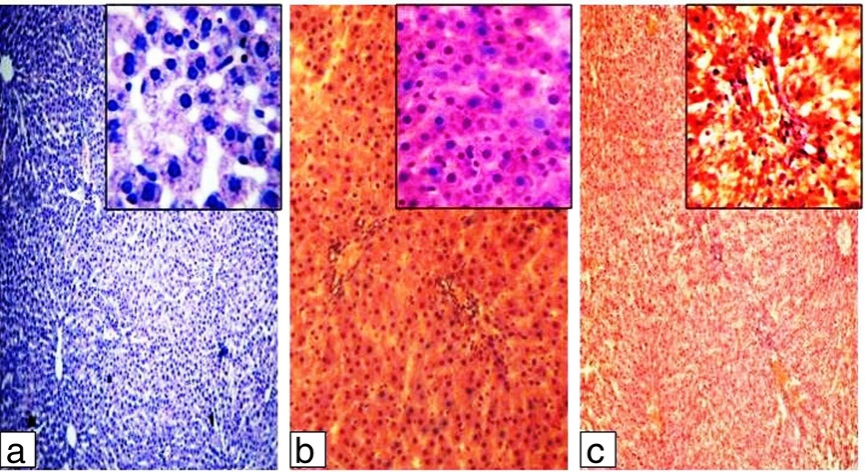
**H & E staining on liver tissue of (a) Control group, provided with normal drinking water, (b) T**_**1**_ **group, provided with 3 days oral intake of** ***N. oleander*** **extract and (c) T**_**2**_ **group, provided with 7 days oral intake of N. oleander extract.** Microphotographs were taken at 100X & 1000X magnifications.

In the Con group, the liver was free from any pathological abnormality, and H&E stained sections appeared normal with regular cellular architecture. The hepatic cells had intact cytoplasm, sinusoidal spaces, prominent nucleus and nucleolus (Figure 3a). Analysis of T_1_ group revealed noticeable infiltration of inflammatory cells likely the lymphocytes around the hepatic arteries & central vein with low level of vascular damage and individual cell necrosis (Figure 3b). In the T_2_ group, relatively increased proportion of binucleated cells in periportal area and inflammatory cells in perivascular area was found, indicating trigger of immune response of the organisms. Hepatic necrosis, widening of sinusoidal spaces and mild level of vascular damage was also observed in H & E stained sections of liver tissue (Figure 3c).

## Discussion

*N. oleander* is considered as poisonous plant. It contains components such as oleandrin and neriine that cause damage by inhibiting pasmalemmal Na^+^, K^+^-ATPase [11]. In the present study the hematological and histopathological alterations were observed after *N. oleander* leaves extract administration in wistar rats. Hematological analysis indicates elevated level of RBCs, Hb, Hct and MCV in experimental animals. Increased number of red blood cells is an indication of polycythemia. In polycythemia, the levels of Hb, Hct, & RBCs are elevated when measured in the complete blood count (CBC), as compared to normal [25]. Erythropoiesis occurs in the bone marrow. Erythropoietin (Epo) is one of the important hormones regulating this process [26]. The majority of Epo is produced and released by kidneys (90%) and liver (10%) [27, 28]. Effect of extract on liver and kidney disturb the Epo expression directly or by disturbing other factors which are responsible for Epo expression. Under toxic conditions kidney release too much of protein (Epo) that enhance RBCs production. RBCs increase enhances the chance of clot formation, which can cause a heart attack or stroke. RBCs elevation is directly related to increase in Hb level [29]. Hct, Packed cell volume is the volumetric content of the RBCs in the blood [30]. Hct contents in the blood have been increased quit significantly (10% & 16%) in experimental groups; Hct can be increased in various physiological conditions such as in Dengue Shock Syndrome, polycythemia, myeloproliferative disorders, COPD chronic obstructive pulmonary diseases and hypoxia [31, 32]. The increase is in erythropoietin produced from the kidneys (specifically) is also the cause in increasing the Hct contents of the blood [33]. The erythropoietin is also been designated as a positive acute phase protein due to its extremely high up regulation in initial stages of Acute phase response [34]. The possible cause of increased Hct in the present study is increase in the MCV. The increase in MCV itself results in relative decrease in plasma [35]. 20% increase in the MCV in T_1_ group blood analysis indicates a pathological state called as macrocytosis. The potent causes of the state are liver disease, compensated hemolysis, or folate deficiency [36].

Neutrophils and its derived cytokines play an essential part in the development and manifestation of inflammation. The stimulation of neutrophils can lead to the generation of oxygen derived free radicals also called reactive oxygen species (ROS) that cause further cellular damage. The formation of free radicals and cytotoxic oxygen metabolites probably impart a key role in various types of tissue degeneration and pathology such as aging, cancer and retinal degeneration [37, 38]. In the present study, significant elevation in the neutrophil count (neutrophilia) occurs with 357% rise in its level in T_2_ group as compared to control was may be due to the free radicals resulting from *N. oleander* leaves extract administration which caused liver injury and a proportion of these free radicals librated into the blood may also affect the circulating cells and induced a significant change in their number [39]. Elevated number of lymphocytes secretes different derivatives. IL-5, a derivative of lymphocytes is responsible for the stimulation and activation of eosinophils [40]. 47% increase in monocytes count indicates a state of stress response, necrosis, red cell regeneration and sarcoidosis [41, 42].

Tissue inflammation performs a crucial job in liver pathology. Experimental groups show infiltration of immune cells, widening of sinusoidal spaces, elevated level of binucleated cells and individual cell necrosis. It has been well established that free radicals elicit an inflammatory response at the site of injury [43]. Toxic elements may induce pro-inflammatory cytokines by activating Kupffer cells [44]. TNF-α, an important pro-inflammatory cytokine that is noticeably concerned in the progression or initiation of inflammatory response [45] and also stimulate the triggering of inflammatory mediators like IL-1, IL-6, TGF-β and prostaglandins that facilitate cellular infiltration, vacuolation and necrosis in the liver [46, 47].

## Conclusions

Taken together these findings we can conclude *that N. oleander* leaves extract significantly affects on hematological and histopathological parameters due to its toxicity. Efforts must be exerted to purify different chemical components from extract with no inflammation as this plant is utilized in folk medicine with narrow therapeutic indices. Seeing that, its use is risky and should be cautiously researched.
